# Impact of Urinary Incontinence on the Quality of Life After Open Retropubic Radical Prostatectomy

**DOI:** 10.7759/cureus.28106

**Published:** 2022-08-17

**Authors:** Yassine Ouanes, Amine Hermi, Kays Chaker, Mokhtar Bibi, Kheireddine Mrad Daly, Yassine Nouira

**Affiliations:** 1 Urology, La Rabta University Hospital, Tunis, TUN

**Keywords:** urinary urgency, quality of life, urinary incontinence, prostatectomy, prostatic neoplasms

## Abstract

Introduction

Radical retropubic prostatectomy is one of the standard treatments for localized prostate cancer. Evaluating the severity of postoperative urinary incontinence is primordial to guiding the treatment choice, and it still lacks standardization, hence the value of assessing the quality of life.

Methods

We conducted a retrospective study between January 2014 and December 2018 in the Urology Department of La Rabta Hospital, involving 30 patients followed for localized prostate cancer treated with radical prostatectomy for at least one year. Our work aimed to study urinary incontinence after radical retropubic prostatectomy and to evaluate the quality of life of patients who underwent surgery for localized prostate cancer by three validated questionnaires: The International Prostate Symptom Score (IPSS), The International Consultation on Incontinence Questionnaire Short Form (ICIQ-SF) and the International Continence Society (ICS) scores.

Results

The preoperative IPSS score ranged from 5-22, averaging 11.13. After the surgery, it ranged between four and 23, with an average of 14.13. This increase was significant, with p = 0.001. The average preoperative ICIQ-SF score was 10.03, and the mean postoperative score was 14.23. The first question dealing with the frequency of episodes of urinary leakage has not demonstrated variation after surgery. In the second question, which deals with the amount of urine loss, we found a significant increase in this parameter with p=0.003. In the third question inherent to perceived discomfort, operated patients reported significant deterioration with p <0.001.

We observed an increase in patients with urinary stress incontinence and enuresis on the ICS score after radical retropubic prostatectomy. Wearing protection or padding was required in 23.3% of patients.

Conclusion

IPSS, ICIQ-SF, and ICS scores are helpful to perform before and after radical retropubic prostatectomy. It helps to study urinary incontinence better, propose to each patient with postoperative complications the appropriate treatment option, and improve the quality of the urinary status.

## Introduction

Prostate cancer is the second most frequent male cancer and the fifth leading cause of death worldwide [1]. Radical prostatectomy (RP) is the standard surgical treatment for localized prostate cancer [2]. This type of surgery can affect the patient physically and mentally, given its side effects that could significantly impact the patient's quality of life (QOL), such as urinary incontinence (UI). Assessing the impact of this side effect on the QOL is primordial for its management. Many subjective and objective tools such as questionnaires are recommended [3]. This series highlights this point by analyzing the incidence of UI after RP and its impact on the QOL.

## Materials and methods

**Figure 1:**
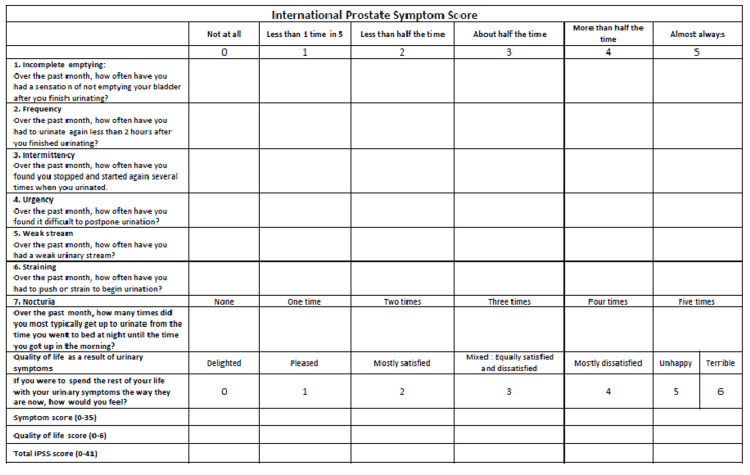
International Prostate Symptom Score [4]

**Figure 2:**
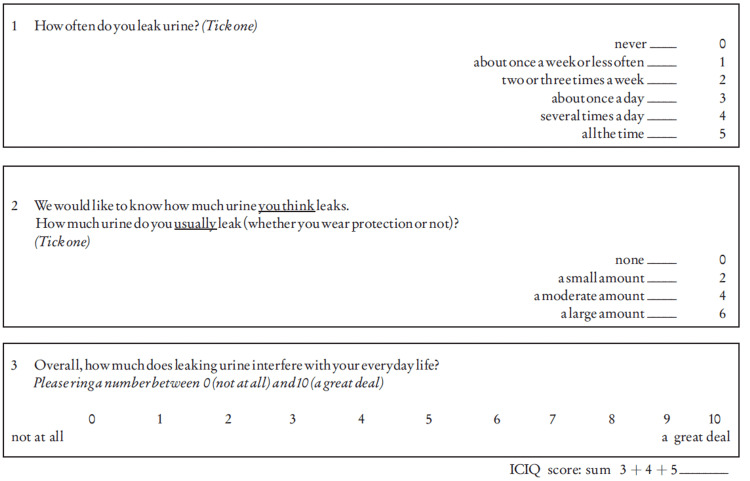
International Consultation on Incontinence Questionnaire Short Form score [5]

**Figure 3:**
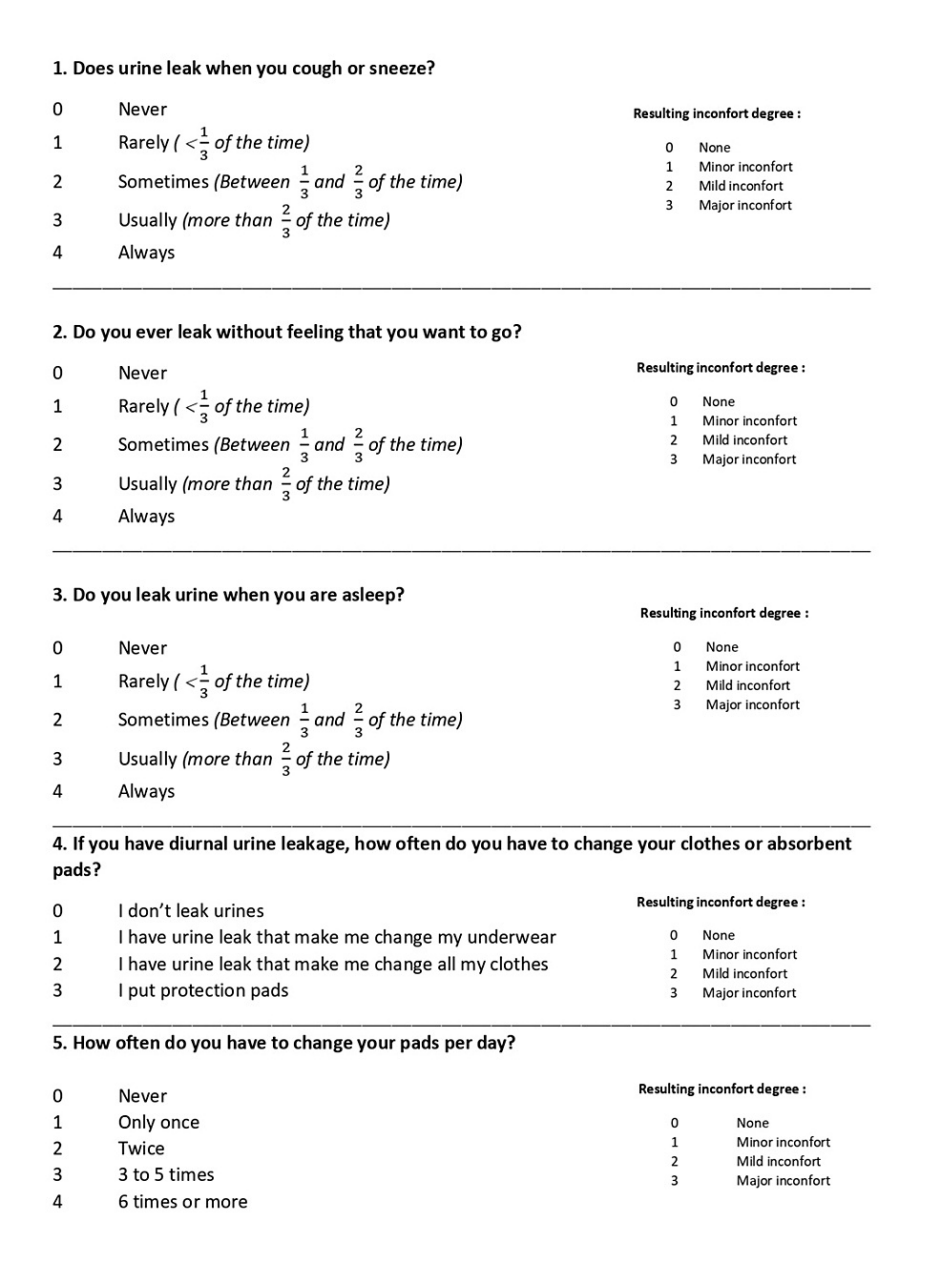
International Continence Society score [6]

This study was a prospective analysis of 30 patients who underwent retropubic RP from January 2014 to December 2018 for localized prostate cancer. The follow-up limit was fixed to at least one year. All patients were advised pelvic floor exercises. The impact of UI on the QOL of the patient was assessed through validated scoring systems, namely, the International Prostate Symptom Score (IPSS) (Figure 1) [4], the International Consultation on Incontinence Questionnaire Short Form (ICIQ-SF) (Figure 2) [5], and the International Continence Society (ICS) scores (Figure 3) [6].

These scores were filled prior to RP and one year after surgery. The interval between completing the questionnaire and surgery ranged from 12 to 28 months, with a mean of 14 months. The exclusion criteria were as follows: (1) patients with baseline severe UI related to neurogenic bladder, synchronous bladder tumor, and history of previous treatment of prostate cancer other than RP, for instance, radiotherapy. Patients with high D'Amico risk prostate cancer and locally advanced prostate tumor (T3, T4, and N+ at MRI) were also excluded. Data were processed and analyzed using the Statistical Package for Social Science, version 25.0 for Windows. Means, medians, and percentages were used to describe the distribution and central tendency of the data. The significance level was fixed at 0.05 (significant if P < 0.05).

## Results

This study enrolled 30 patients who underwent open RP. Patients ranged in age between 52 to 71 years, with a mean of 64 years. Prostatic cancer was diagnosed on resected tissue in two [A1] patients (6.7%) and by transrectal biopsy in the remaining 28 cases (93.3%). Four patients (13.3%) underwent bilateral nerve-sparing surgery in case of a localized prostatic tumor. Histological examination of the RP specimens showed that the cancer was limited to the prostate in 26 patients and was extracapsular in four cases. Surgical margins were negative in 24 patients (80%) and positive in 6 cases (20%). Pelvic lymph node dissection was performed in 19 cases, with positive histological findings in 89.4% of the cases (17 patients). Postoperative follow-up showed that vesicourethral anastomotic stenosis occurred in four cases (13.3%), treated by internal urethrotomy. The median IPSS scores for the patients preoperatively and at one year after surgery were 11.13 (from 5 to 22) and 14.3 (from 4 to 23), respectively, which increased significantly by 3.34 (P = 0.001). The ICIQ-SF score assessed patients. Before surgery, the mean value was 10.3, ranging from 6 to 15. This score climbed to 14.23 postoperatively (8-20). As explained in Table 1, patients reported a statistically significant increase in the leakage volume after surgery (Question 2) and a significant deterioration of the overall QOL related to UI (Question 3). No difference was observed in the frequency of UI (Question 1).

**Table 1:** Results of the ICIQ-SF score and its statistical significance for each question ICIQ-SF: International Consultation on Incontinence Questionnaire Short Form

	Preoperative	Postoperative	P value
Q1: The first question's mean score	2.7	2.7	0
Q2: The second question's mean score	4.26	5.63	0.003
Q3: The Third question's mean score	3.06	6	< 0.001

The ICS score revealed that UI and enuresis were more important after RP. Absorbents pads were used postoperatively by 23.3% of the patients.

As shown in Table 2, responding to the first question in the ICS score, "Does urine leak while sneezing or coughing?" half of the patients reported preoperative UI, with minor impact on the QOL. Eleven patients had a moderate impact on QOL (36.7%) after surgery versus only one patient preoperatively.

**Table 2:** Results of the first question in the ICS score: Does urine leak while sneezing or coughing? ICS: International Continence Society

	Attributed score	Number of patients	
		Pre-operative (%)	Post-operative (%)
Question 1	1	15 (50%)	0
	2	14 (46.7%)	6 (20%)
	3	1 (3.3%)	24 (80%)
Inconfort degree	0	15 (50%)	5 (16.7%)
	1	14 (46.7%)	14 (46.7%)
	2	1 (3/3%)	11 (36.7%)

The results of answering the second question in the ICS score, "Do you have an involuntary loss of urine without a sense of urgency?" showed that only one patient claimed to have significant involuntary urine loss preoperatively. After surgery, this complaint was present in 18 patients (60%). Significant discomfort was reported after surgery in 19 patients (63.3%) (Table 3).

**Table 3:** Results of the second question in the ICS score: Do you have an involuntary loss of urine without a sense of urgency? ICS: International Continence Society

	Attributed score	Number of patients	
		Pre-operative (%)	Post-operative (%)
Question 2	1	15 (50%)	1 (3.3%)
	2	14 (46.7%)	11 (36.7%)
	3	1 (3.3%) *	18 (60%) *
Inconfort degree	0	15 (50%)	0
	1	15 (50%)	11 (36.7%)
	2	0	19 (63.3%) **

For the answers to the third question of the ICS score, "Do you have a nocturnal urinary loss?" seven patients (23.3%) reported major enuresis de novo, with significant discomfort degree (Table 4).

**Table 4:** Results of the third question in the ICS score: Do you have a nocturnal urinary loss? ICS: International Continence Society

	Attributed score	Number of patients	
		Pre-operative (%)	Post-operative (%)
Question 3	1	7 (23.3%)	0
	2	23 (76.7%)	23 (76.7%)
	3	0	7 (23.3%) *
Inconfort degree	0	9 (30%)	0
	1	21 (70%)	12 (40%)
	2	0	18 (60%)

Concerning the fourth question of the ICS score, "If you have diurnal urine leakage, how often do you have to change your clothes or absorbent pads?" no patients have reported the need for absorbent pads before surgery. Postoperatively, seven patients began to use pads, and, as a consequence, a significant increase in the sense of discomfort, from 46.7% before surgery to 80% after surgery, was observed (Table 5).

**Table 5:** Results of the fourth question in the ICS score: If you have diurnal urine leakage, how often do you have to change your clothes or absorbent pads? ICS: International Continence Score

	Attributed score	Number of patients	
		Pre-operative (%)	Post-operative (%)
Question 4	1	6 (20%)	0
	2	24 (80%)	23 (76.7%)
	3	0	7 (23.3%)
Inconfort degree	0	15 (50%)	0
	1	1 (3.3%)	6 (20%)
	2	14 (46.7%)	24 (80%)

For the results of the fifth question in the ICS score, "How often do you have to change your pads per day?," after surgery, 23 patients (76.7%) used from three to five pads per day, whereas seven patients needed to change their pads from three to four times during nighttime (table 6).

**Table 6:** Results of the fifth question in the ICS score: How often do you have to change your pads per day? ICS: International Continence Society

	Attributed score	Number of patients	
		Pre-operative (%)	Post-operative (%)
Question 5	1	15 (50%)	0
	2	15 (50%)	23 (76.7%)
	3	0	7 (23.3%)
Inconfort degree	0	15 (50%)	0
	1	15 (50%)	23 (76.7%)
	2	0	7 (23.3%)

## Discussion

Despite continuous surgical advances, changes in urinary function occur inevitably after RP, with stress UI being a frequent adverse effect reported in 20% to 87% of cases, depending on the definition used and the timepoint assessed [2,3]. In most cases, the UI resolves within eight months [7,8]. The most common cause of post-prostatectomy incontinence is external sphincter dysfunction, which is present in 88% of cases through direct injury, neuropraxia, or supporting structures [2,3]. However, it often coexists with detrusor instability [3]. It is common and major morbidity that can affect the QOL of patients. To date, no standardized method has been established to assess the impact of UI on the QOL of patients after prostatectomy. Various questionnaires are used for the subjective evaluation of UI, for instance, the IPSS and ICIQ-SF questionnaire and the ICS scoring system.

To some extent, these different methods have shown correlations among their results that vary widely. This paper evaluated UI using IPSS, ICIQ-SF, and ICS scores before and after RP. The IPSS questionnaire, also known as the AUA Symptom Score, was designed to be easily self-administered by the patient [4]. It has been used to assess lower urinary tract symptoms (LUTSs) related to prostatic hyperplasia [9,10]. However, some studies [4,5] reported its usefulness in patients who underwent prostatectomy for prostate cancer. In our study, the mean IPSS scores for patients preoperatively one year after surgery were 11.13 and 14.3, respectively. It has increased significantly by 3.34 (P = 0.001). 

Lorion et al. [8] evaluated patients treated for localized prostate cancer retrospectively. No changes were noted in voiding quality after five years, as assessed by the IPSS score [4]. The analysis demonstrated a comparable evolution for total prostatectomy, external radiotherapy, and brachytherapy, with an early deterioration in the IPSS scores and associated QOL, with a return to baseline scores 12 months after treatment and even an improvement in scores at five years [4]. The ICIQ-SF score is a brief and straightforward UI questionnaire. It was developed and validated in 1998 to assess the impact of UI on the quality of everyday life [5]. In our series, the ICIQ-SF score showed a statistically significant increase in the volume of urine loss and the inconvenience sense secondary to urinary loss one year after radical surgery. The ICIQ-SF score also assessed patients. Before surgery, the mean value was 10.3, ranging from 6 to 15. This score climbed to 14.23 postoperatively (8 to 20). Patients reported a significant increase in the leakage volume after surgery and a significant deterioration of the overall QOL related to UI. No difference was observed in the frequency of urinary loss. Machioka et al. [10] reported a maximum preoperative ICIQ score of 11 points, matching with a maximum pad weight of 31 g per day. The mean postoperative ICIQ-SF showed different variations, that is, 0, 10, 7, 5, and 4 at 1, 3, 6, and 12 months. The total ICIQ-SF score returned to its preoperative level twelve months after surgery in 67% of patients and 64% on the 24-h pad weight test [8]. The authors pointed out that the probability of UI differed according to how it was defined; that is, the continence rate 12 months after RP was 67% for no pad use, 87% for one security pad per day, and 94% for one pad per day. The proportions of no pad use at 1, 3, 6, and 12 months after RP was 12%, 32%, 52%, and 67%, respectively. 

The three evaluation methods (24-h pad weight, daily pad use, and ICIQ-SF score) showed recovery to the preoperative level for 29% of patients [8]. Previous reports [9] certified that the higher the patients’ ICIQ score before RP, the greater the number of patients who recovered the same or better UI status after surgery. Patients dissatisfied with their urinary status before surgery felt more tolerant toward it after RP. On the other hand, patients satisfied with their urinary status before treatment found it unacceptable after RP, even if at an insignificant level [8]. The UI, as defined by the ICS, does not involve the criteria of frequency, urinary volume, or pad use; however, it offers a vital role in the personal evaluation of the symptom incontinence and its impact on the patient’s QOL. Our study made it possible to assess UI as defined by the ICS of the patients after surgery precisely. We found that most of them presented some degree of incontinence, significantly impacting the QOL. Absorbents pad two to four times a day postoperatively by 23.3% of the patients. In this study, the authors found no benefit in preferring the ICS SF to the IPSS. However, in their study population, the rate of UI was low (3.7% of patients), which may explain this result.

On the other hand, after RP, the incontinence rate (all grades combined) at two years varied from 10% to 15% [10]. The ICS male SF, therefore, seems to have its place, at least combined with the IPSS. In his study, Erauso [9] prospectively studied the evolution of post-surgery continence in 300 patients. Three months after surgery, 78.5% of patients needed zero to one protection pad per day, and 97.5% needed it for one year. Thus, these results seem superior to those of our study. Most studies note that the UI rate is likely to vary widely 12 months after surgery [8]. Some studies [10-14] identified preoperative factors that might identify patients at risk for UI after RP. For instance, in men aged 65 years or older, the baseline pad weight was more than 8 g and previous TURP. In light of these findings, patients can be given more realistic expectations regarding the risk of complications after a total prostatectomy. This point is essential for fully informed consent. Furthermore, adequate preoperative counseling reduces the impact of UI on the QOL [11]. This study assessed the utility of combining three subjective scores to evaluate incontinence severity and its impact on QOL.

Significant correlations were found between these scores with different degrees. The strongest correlation concerned the incontinence frequency with ICIQ-SF and ICS, as shown in tables 1, 2. The IPSS is not primarily designed to assess UI. However, it showed that RP does not necessarily bring benefits to the pre-existing LUTS. These data further strengthen the construct validity of these scores, and clinicians can be confident in its use in assessing postoperative UI. Our study, however, presents some limitations. It was based on data from only a 1-year follow-up after surgery, whereas a longer-term follow-up might affect UI status more unfavorably because of patients’ aging. Thus, a longer follow-up period would be helpful. Moreover, the study population was small, and accumulating a more significant number of cases would be necessary to strengthen our results.

## Conclusions

UI is an important consideration for patients proposed to have RP, given that it might have a significant impact on the QOL. Thus, urologists must help patients decide on their treatment choice based on the proposed treatment's relative merits and the likely complications. In this study, IPSS, ICIQ-SF, and ICS scores showed reliability and simplicity in assessing the impact of UI on the patient's QOL. Associating these scores with other validated objective evaluation means such as the 24-pad weight can contribute to a more precise evaluation. Further studies with a longer oncological and functional outcomes follow-up are needed to manage this complication better.
